# Trends in Endometrial Cancer in Poland: Shifts in Clinical Features and Survival Outcomes over 18 Years

**DOI:** 10.3390/jcm14020566

**Published:** 2025-01-17

**Authors:** Marcin Misiek, Grzegorz Witczak, Agnieszka Picheta, Michał Skuza, Aleksandra Misiek, Tomasz Kluz, Andrzej Wróbel, Anita Chudecka-Głaz

**Affiliations:** 1 Department of Gynecologic Oncology, Holy Cross Cancer Center, 25-734 Kielce, Poland; 2 Department of Gynecological Surgery and Gynecological Oncology of Adults and Adolescents, Pomeranian Medical University, 70-204 Szczecin, Poland; grzegorzwitczak9@gmail.com (G.W.);; 3 Institute of Medical Sciences, Jan Kochanowski University, 25-369 Kielce, Poland; 4 Department of Gynecology and Obstetrics, Institute of Medical Sciences, Medical College of Rzeszow University, 35-310 Rzeszow, Poland; 5 Second Department of Gynecology, Medical University of Lublin, Jaczewskiego 8, 20-954 Lublin, Poland

**Keywords:** endometrial cancer, overall survival, trends, clinical outcomes

## Abstract

**Background/Objectives:** Endometrial cancer is becoming an even more significant health concern in Poland, with incidence and mortality rates rising each year. **Methods**: This retrospective study analyzed 1532 patients surgically treated for endometrial cancer at a single center in Poland between 2002 and 2020, examining changes in clinical and histopathological characteristics and their impact on patient outcomes over three time periods: 2003–2008, 2009–2014, and 2015–2020. **Results**: The study revealed significant shifts in tumor characteristics over time. Early-stage tumors (FIGO IA) increased in prevalence, from 34.1% in 2003–2008 to 49.8% in 2015–2020 (*p* < 0.001), while advanced-stage cases (FIGO IIIC or higher) decreased from 12.1% to 8.1% (*p* < 0.001). Similarly, well-differentiated tumors (G1) rose from 46.5% to 62.6% (*p* < 0.001), while poorly differentiated tumors (G3) decreased slightly from 13.4% to 12.2%. Histologically, the incidence of most typical endometrioid carcinoma peaked at 92.6% in 2009–2014 with 77.4% in 2015–2020 (*p* < 0.001). The prevalence of serous carcinoma significantly decreased from 16.5% in 2003–2008 to 1.2% in 2009–2014 and 3.2% in 2015–2020. **Conclusions**:Statistically significant differences in overall survival (OS) across the time periods were found. Three-year OS was 78.0% for patients treated in 2003–2008, compared to 66.2% in 2009–2014 and 69.9% in 2015–2020 (*p* = 0.024). Similarly, 5-year OS was significantly higher for the 2003–2008 group at 68.8% compared to 50.2% for the 2009–2014 group (*p* = 0.001). However, progression-free survival (PFS) did not differ significantly at either the 3-year (*p* = 0.279) or 5-year (*p* = 0.279) time points.

## 1. Introduction

Endometrial cancer is a global health issue. It is estimated to account for approximately 76,000 deaths of women annually worldwide [1]. In Poland, the incidence of uterine cancer is estimated at 7.7% [2]. Globally, studies in 160 countries have shown that age-standardized incidence rates (ASIR) have increased significantly in the last 30 years [3]. According to GLOBOCAN data, not only the incidence but also the mortality rates have risen in Poland, as well as in many developing countries, in the last decades [4]. The increasing number of cases each year poses diagnostic, therapeutic, and organizational challenges for the healthcare system.

Regarding the basic division proposed by Bokhman, type I endometrial cancer—endometrioid carcinoma—is associated with prolonged stimulation of the endometrial lining by estrogens such as estradiol (E2), which is the dominating estrogen during the pre-menopausal period, and estrone, which is a crucial hormone during the postmenopausal years of a woman’s life. The effect of estrogen hormones on the endometrium is achieved through their binding to estrogen receptors (ERα and ERβ) on target cells, initiating a cascade of gene expression that promotes cell proliferation, tissue growth, and preparation for potential implantation. The counterbalancing effect of progestogens is achieved by the downregulation of estrogen receptors (ER) as well as by promoting differentiation of the cells of the endometrium [5,6]. Type II tumors—non-endometrioid carcinomas—are usually not related to this hormonal imbalance, exhibiting different development dynamics and significantly worse prognosis. Type II includes cancers such as serous carcinoma, clear cell carcinoma, undifferentiated tumors, and carcinosarcomas. This classification is also justified genetically. Long-term genetic studies have shown that the cause of most cases of endometrioid cancer is the inactivation of the PTEN tumor suppressor gene [6]. For serous cancers, the majority involvement is the inactivation of the TP53 tumor suppressor gene. The development of molecular and genetic research techniques has enabled the identification of endometrial cancer classification based on the genetic background during the work on The Cancer Genome Atlas (TCGA). Four subgroups were distinguished: POLE, MSI (microsatellite instability), Copy Number Low (CNL), and Copy Number High (CNH) [7]. This classification indicated that categorizing tumors solely based on histopathological type was insufficient for appropriate prognostic stratification, thereby affecting the planning and implementation of suitable treatment [8,9]. The percentage of various types of tumors likely changed over time, which may be related to environmental factors, lifestyle changes in societies, and various other epigenetic factors.

Therapeutic approaches have also evolved, partly due to the increasing importance of minimally invasive surgery in the treatment of endometrial cancer [10].

In this study, we aimed to assess whether statistical prognostic indicators, as well as clinical factors such as grading and histological types of endometrial cancer, have changed and influenced the outcome of the disease over the past 18 years.

## 2. Materials and Methods

This retrospective single-center study was carried out at the Department of Gynecologic Oncology, Holy Cross Cancer Center, in Kielce, Poland. It included 1532 patients diagnosed with endometrial cancer and treated surgically at the department from 2002 to 2020. The study adhered to the Declaration of Helsinki and received approval from the Ethics Committee of Jan Kochanowski University in Kielce, Poland (protocol code 39/2023, approved on 8 September 2023). Data for the study were retrospectively collected from hospital medical records and pathology reports. The results of histopathological examinations that were included in the study were final results, concluded after surgeries, and did not include results that were reported at the first stage of diagnosis which included hysteroscopies or dilatation and curettage procedures. MRI (Magnetic Resonance Imaging), CT scans (Computer Tomography), and histopathological examination reports acquired after surgical treatment were diagnostic tools used in assessing the FIGO stage of the disease. While the data collection for analysis in the study was finished in 2020, we have chosen to follow FIGO (2009) staging instead of the most up-to-date FIGO (2023) staging system.

Statistical analyses were performed in R software, version R-4.1.2. Numerical variables were described with mean and standard deviation or median and interquartile range, depending on the distribution. Nominal traits were summarized with absolute frequency and proportion. A distribution normality check was performed with the Shapiro–Wilk test and verified with skewness and kurtosis, while homogeneity was assessed with the Levene test. Comparisons were made using the Student *t*-test for independent groups, Mann–Whitney U test, Pearson chi-square test, or Fisher exact test, as appropriate. Correlation analysis was performed with the Spearman method. Survival analysis was run using the Kaplan–Meier method and log-rank test for survival differences between groups. The proportional hazard Cox regression model was employed to quantify the impact of selected variables on survival. Regression analysis was run in 2 steps: univariate and multivariate. The final shape of the multivariate model was based on stepwise variable selection.

All tests assumed alpha = 0.05.

## 3. Results

### 3.1. Study Group Characteristics

The total number of patients was 1532, with an average age of 64.31 ± 9.99 years. Less than half of the patients were at FIGO stage IA (43.2%), 18.7% were at stage IB, and 20.5% were at stage II. Analysis of histological grading revealed that 50.8% of the patients were G1, 34.8% were G2, and 13.3% were G3. Endometrioid adenocarcinoma was identified in 88.8% of the patients. Majority of patients had type I cancer (89.5%), while type II was observed in 9.6%. Lympho-vascular space invasion was present in 17.5% of the cases.

Surgeries were performed between 2002 and 2020. We decided to divide the study period into three 6-year intervals to assess possible changes over time. The first period (2003–2008) included 23.8% of patients, the second period (2009–2014) included 34.6%, and the third period (2015–2020) included 39.8%. We divided surgical procedures into those that were performed via laparotomy (Total Abdominal Hysterectomy (TAH)) and laparoscopic procedures (total laparoscopic hysterectomy (TLH)). Lymphadenectomy, if necessary and possible, was performed in both types of procedures. Most surgeries were performed via laparotomy (72.1%). Blood transfusions were required in 4.3% of procedures. Reoperation was necessary for five patients. Recurrence occurred in 6.7% of cases. Over 22.5% of patients survived at least five years, while 30.3% died during the analysis period. Systemic lymphadenectomy was performed in 65.7% of the operations, while the rest of the group underwent a sentinel lymph node biopsy procedure or was not qualified for any kind of lymphadenectomy. Detailed information is presented in Table 1.

The population of patients described in Table 1 was also described by Misiek et al. in an article regarding a retrospective study analyzing the implications of pelvic lymphadenectomy [11].

### 3.2. Histological Type

There was a significant difference in HP structure between the analyzed periods (*p* < 0.001). The incidence of endometrioid adenocarcinoma was 72.7%, 92.6%, and 77.4% starting from the earliest and ending with the latest period. Moreover, the serous type was seen visibly more often in 2003–2008 (16.5%) compared to 2009–2014 (1.2%, n = 2) and 2015–2020 (3.2%). The incidence of other HP types increased in the last period (17.1%) compared to the first two analyzed periods (6.6%, n = 8, 4.3%, n = 7).

### 3.3. FIGO Staging

FIGO stage structure differed depending on the period, *p* < 0.001. The incidence of FIGO IA was the lowest in 2003–2008 (34.1%) and grew in the next two periods (42.6% and 49.8% in 2009–2014 and 2015–2020, respectively). The incidence of FIGO IB was the highest in 2003–2008 (27.5%) and decreased in the next periods (18.0% and 13.9% in 2009–2014 and 2015–2020, respectively). Additionally, a lowering proportion of FIGO IIIC or higher was observed over time (12.1%, 11.2%, and 8.1%, from the earliest to the latest period).

### 3.4. Grading

Grading structure differed significantly between periods, *p* < 0.001. G1 was observed more often in 2015–2020 (62.6%) compared to the two earlier periods (46.5% and 40.2% in 2003–2008 and 2009–2014, respectively). The proportion of G2 was the lowest in the last period (23.6%), compared to the first two periods (39.2% and 44.9% in 2003–2008 and 2009–2014, respectively). The frequency of type I cancer was not significantly different depending on the period, *p* = 0.137. The frequency of type II cancer was significantly different depending on the period, *p* = 0.014, with the highest proportion in 2009–2014 (12.5%) and a lower proportion in the other two periods (8.5% and 7.5% in 2003–2008 and 2015–2020, respectively) (Table 2).

### 3.5. Survival Analysis

OS and PFS times among three groups of patients in 2-year and 3-year periods were assessed. A 5-year analysis of PFS and OS was performed for groups that were treated between 2003–2008 and 2009–2014, which were chosen to analyze the most adjusted data.

#### 3.5.1. OS and PFS Analysis in 2-Year Period

Median 2-year survival was not reached in any of the groups.

**Figure 1:**
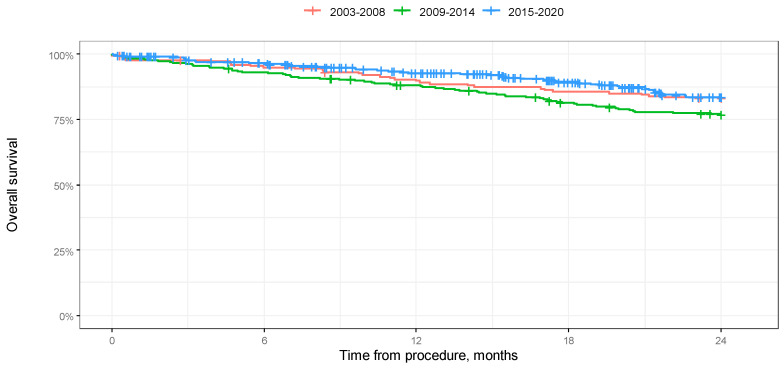
Kaplan–Meier overall survival curves depending on year of procedure in 24-month-long observation.

**Figure 2:**
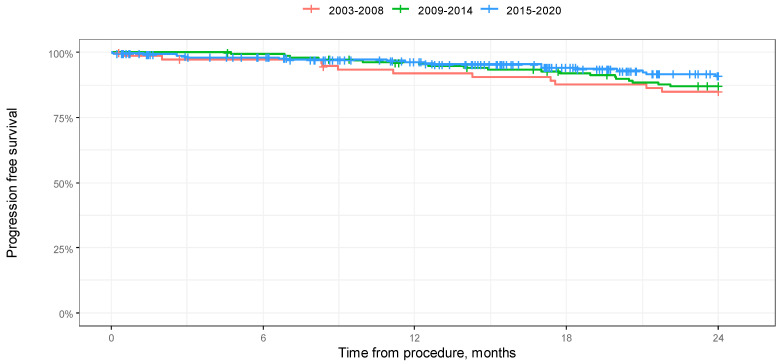
Kaplan–Meier progression-free survival curves depending on year of procedure in 24-month-long observation.

According to a log-rank test, the difference in 2-year overall survival between the groups was not significant (*p* = 0.051). Median 2-year progression-free survival was also not reached, and according to the log-rank test, the difference in 2-year progression-free survival between the groups was not significant (*p* = 0.333). The results of the analysis in the form of Kaplan–Meier curves are shown in Figure 1 and Figure 2.

#### 3.5.2. OS and PFS Analysis in 3-Year Period

The next step was the analysis of OS and PFS for all the groups of patients but including a longer period of 36 months of observation. In this case, statistically important differences between the groups were found.

Median 3-year survival was not reached in any of the groups.

**Figure 3:**
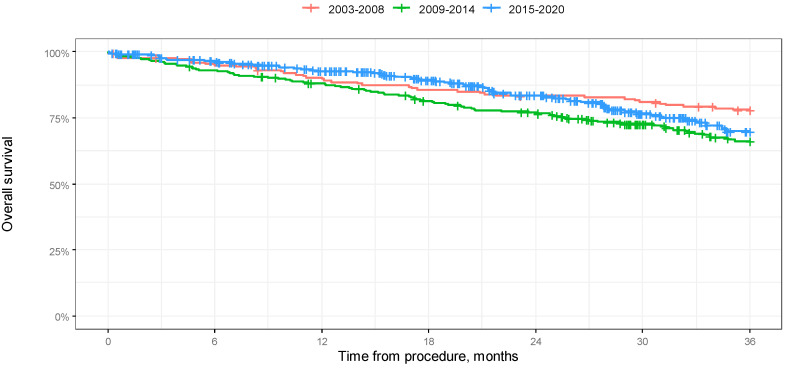
Kaplan–Meier overall survival curves depending on year of procedure in 36-month-long observation.

**Figure 4:**
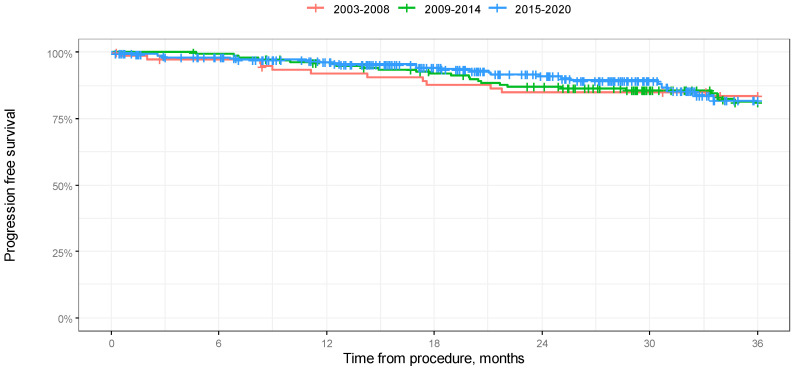
Kaplan–Meier progression-free survival curves depending on year of procedure in 36-month-long observation.

According to a log-rank test, the difference in 3-year overall survival between the groups was significant (*p* = 0.024). As a result of 3-year OS analysis, we found that the group treated between 2003 and 2008 had significantly higher survival rates: 11.8% in comparison to the group treated between 2009 and 2014 and 8,1% in comparison with the group treated between 2015 and 2020. According to the log-rank test, the difference in 3-year progression-free survival between the groups was not significant (*p* = 0.279). The results of the analysis in the form of Kaplan–Meier curves are shown in Figure 3 and Figure 4. A comparison of OS in 12-, 24-, and 36-month-long periods is presented in Table 3.

#### 3.5.3. OS and PFS Analysis in 5-Year Period

We chose to analyze OS and PFS in a 60-month-long observation for the first two groups only. Median 5-year survival was not reached in both groups. According to a log-rank test, the difference in 5-year overall survival between the groups was significant (*p* = 0.001).

Median 5-year progression-free survival was not reached in any of the groups.

**Figure 5:**
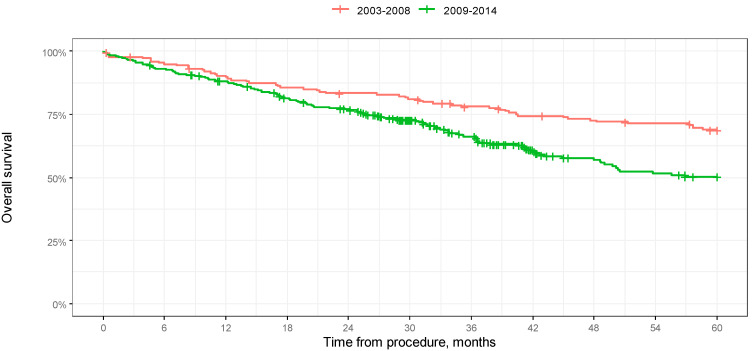
Kaplan–Meier overall survival curves depending on year of procedure in 60-month-long observation.

**Figure 6:**
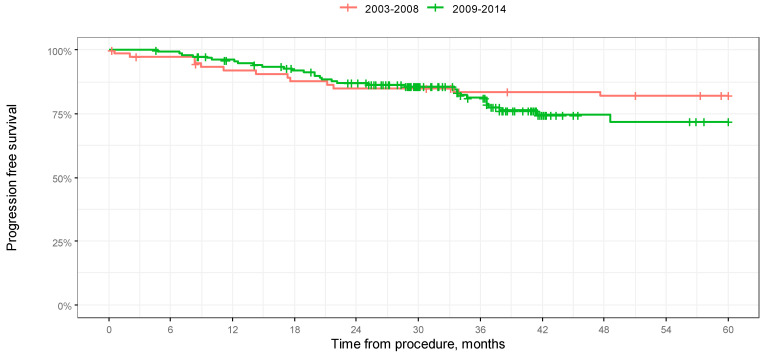
Kaplan–Meier progression-free survival curves depending on year of procedure in 60-month-long observation.

According to the log-rank test, the difference in 5-year progression-free survival between the groups was not significant (*p* = 0.279). The results of the analysis in the form of Kaplan–Meier curves are shown in Figure 5 and Figure 6. A comparison of OS in 12-, 24-, 36-, 48-, and 60-month-long periods is presented in Table 4.

## 4. Discussion

Even though this study was performed at a single oncological center, the results indicate that progress made in the area of operational techniques, as well as development in adjuvant therapy, might not directly translate into better prognosis for patients with endometrial cancer. The fact that Poland has been a rapidly evolving economy in past decades has a huge influence on lifestyle changes that might in some cases have a crucial impact on the incidence rates of certain health-related states. Having in mind those factors, it is understandable that in recent years, Poland has seen a notable increase in the incidence of endometrial cancer. According to data from the Polish National Cancer Registry, the incidence rate has risen steadily, with an estimated 8500 new cases expected in 2024 [12]. Several factors contribute to this trend. The most prominent risk factor is obesity, which is strongly associated with endometrial cancer due to excess estrogen produced by adipose tissue. In Poland, the obesity rate has been climbing, with nearly 25% of women being classified as obese [13]. Women with a body mass index (BMI) of 30 or higher have a two to four times greater risk of developing endometrial cancer compared to those with a BMI under 25. Studies have shown that a 10% increase in BMI resulted in a 9.2% increase in the odds of all-cause mortality [14]. Other contributing factors include diabetes, hypertension, and the use of unopposed estrogen therapy [15]. Additionally, the increasing prevalence of metabolic syndrome, characterized by a cluster of conditions such as high blood pressure, high blood sugar, and abnormal cholesterol levels, correlates with the rising incidence rates. All those factors seem to suggest that patients that will require treatment due to endometrial cancer might be those with multiple diseases and conditions having a substantial impact on treatment outcomes [16,17].

Studies have shown that obese patients tend to have worse OS regardless of cancer type [18].

The preferable operational technique, minimally invasive surgery, has been shown in many studies conducted worldwide to be no less effective and safe regarding OS and PFS than the traditional approach via laparotomy [19,20]. Introducing into clinical use more effective forms of therapy such as PD-L1-oriented immunotherapy for patients with primary advanced and recurrent endometrial cancer will most likely have a visible impact on the results of treatment in those groups of patients [21,22]. Introducing the endometrial cancer classification proposed in the PORTEC-3 trial into clinical use might also result in better qualification of patients for the adjuvant therapy, inducing better treatment outcomes in the future [23].

**Figure 7:**
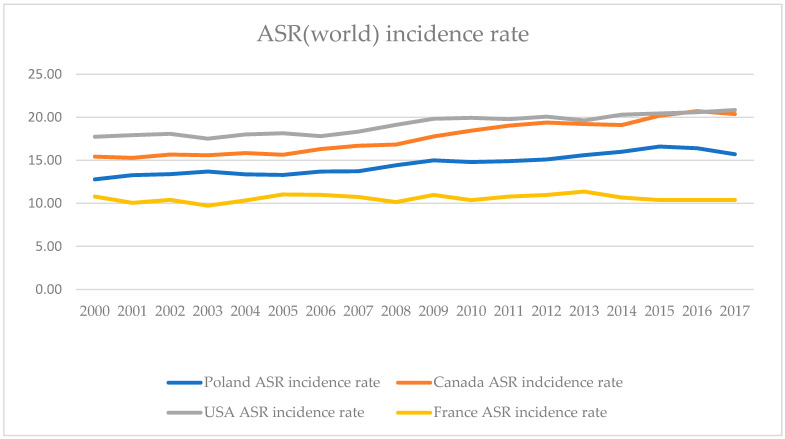
Age-Standardized Rate (World) per 100,000 women, endometrial cancer incidence rate in Poland, Canada, USA, and France 2000–2017.

**Figure 8:**
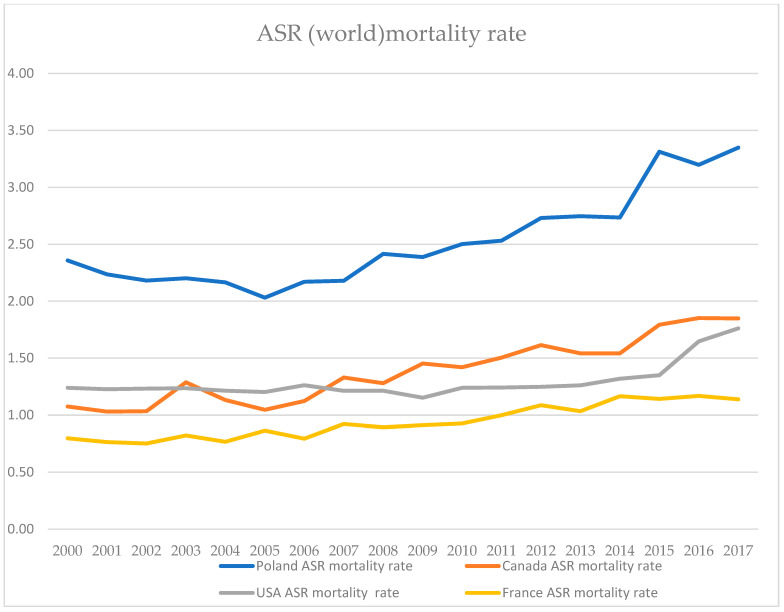
Age-Standardized Rate (World) per 100,000 women, endometrial cancer mortality rate in Poland, Canada, USA, and France 2000–2017.

In order to compare the results of our study with data regarding general trends in endometrial cancer outcomes available in the registers of GLOBOCAN and the Polish National Cancer Registry, we decided to compare incidence rates as well as mortality rates not only in Poland but also in USA, Canada, and France [1,24]. Comparable data were regarding the period from year 2000 to 2017. A deeply concerning trend was observed, especially in Poland and North American countries where mortality rates tend to rise quicker than the incidence rates. The results of that comparison are depicted in Figure 7 and Figure 8.

Comparing the results of our study to the results of an international study EUROCARE-5, the 5-year OS rate, which was 68.8% for the 2003–2008 group in our study, was 2% lower than the 70.8% rate in the general population in Poland in the 2000–2007 period [25]. Such a result might require further investigation but could be possibly explained by the fact that many of the patients without the heavy burden of multiple diseases and obesity were treated in regional hospitals instead of oncological centers.

The increasing detection of low-stage endometrial cancer cases possibly aligns with a growing trend of women attending gynecological check-ups earlier and more regularly, enabling diagnosis at an earlier stage. However, as highlighted in the updated FIGO 2023 staging, even some of these early-stage cancers may fall into high-risk categories due to specific molecular features, such as TP53 mutation. This emphasizes the need for molecular profiling to guide treatment properly, even in seemingly low-risk, early detected cases [26].

Improvement in understanding current endometrial cancer risks and treatment possibilities will possibly require long-term studies, which will incorporate modern genetic approaches to endometrial cancer as a non-homogenous disease.

## 5. Conclusions

This study highlights significant differences in clinical and histopathological features, as well as 2-, 3-, and 5-year survival outcomes, for patients with endometrial cancer based on specific periods of time in the last 18 years. Statistically significant changes in histological type (*p* < 0.001, *p* < 0.001) were observed, with the incidence of endometrial type of cancer being higher in 2009–2014 (92.6%) than in the earlier 2003–2008 (72.7%) and later 2015–2020 (77.4%) periods. A decreasing trend was observed regarding the serous type, which has been most frequent in 2003–2008 (16.5%) compared to subsequent periods (1.2% in 2009–2014 and 3.2% in 2015–2020). Additionally, the incidence of other histopathological types increased in the latest period (17.1%) compared to earlier ones.

Changes in FIGO staging over time were also statistically significant (*p* < 0.001, *p* < 0.001), with an increasing proportion of early-stage (FIGO IA) cases observed over the three periods (34.1%, 42.6%, and 49.8%, respectively) and a decrease in advanced stages (FIGO IIIC or higher) from 12.1% in 2003–2008 to 8.1% in 2015–2020. Furthermore, grading structure varied significantly (*p* < 0.001), with the proportion of G1 tumors being highest in 2015–2020 (62.6%) and lower in earlier periods (46.5% and 40.2%). These results might indicate a time-related tendency to diagnose patients at earlier stages of disease development.

In terms of survival outcomes, statistically significant differences were observed in 3-year and 5-year overall survival (OS). The 3-year OS was significantly better for patients treated between 2003 and 2008 compared to those treated in later periods (*p* = 0.024), and the 5-year OS for the same group was also significantly higher (*p* = 0.001). However, no significant differences were observed in progression-free survival (PFS) between any of the analyzed periods.

These alarming trends require deep analysis not only in Poland but also on the international level. Having in mind that incidence rates of endometrial cancer are a rising trend in many countries, we believe that further investigation is required in many areas including the assessment of genetic, social, and healthcare system factors that might have an impact on outcomes in endometrial cancer treatment in the future.

## Figures and Tables

**Table 1:** Study group characteristics.

Variable	n (%)
Age, years, mean ± SD	64.31 ± 9.99
FIGO(2009)	
IA	653 (43.2)
IB	282 (18.7)
II	310 (20.5)
IIIA	62 (4.1)
IIIB	46 (3.0)
IIIC	3 (0.2)
IIIC1	94 (6.2)
IIIC2	15 (1.0)
IVA	5 (0.3)
IVB	41 (2.7)
Grading	4 (1.8)
G1	754 (50.8)
G2	517 (34.8)
G3	198 (13.3)
Gx	16 (1.1)
HP	
Endometrioid carcinoma	1352 (88.8)
Clear cell carcinoma	31 (2.0)
Mixed carcinoma	29 (1.9)
Serous carcinoma	57 (3.7)
Carcinosarcoma	14 (0.9)
Other	40 (2.6)
Type I	1369 (89.5)
Type II	147 (9.6)
LVSI	266 (17.5)
Procedure timing	
Year 2002	28 (1.8)
Years 2003–2008	364 (23.8)
Years 2009–2014	530 (34.6)
Years 2015–2020	610 (39.8)
Procedure type	
TLH	335 (27.9)
Laparotomy—TAH	867 (72.1)
Blood transfusion	24 (4.3)
Reoperation	5 (1.4)
Relapse	37 (6.7)
5-year survival	234 (87.3)
Death	452 (30.3)
Lymphadenectomy	1004 (65.7)

SD—standard deviation, IQR—interquartile range, TLH—total laparoscopic hysterectomy, TAH—Total Abdominal Hysterectomy, LVSI—lympho-vascular space invasion.

**Table 2:** Clinical feature differences between analyzed groups.

Period	2003–2008	2009–2014	2015–2020	*p*
Variable	n (%)	n (%)	n (%)	
Relapse	17 (15.3)	6 (3.7)	14 (4.9)	<0.001
HP				
Endometrioid carcinoma	333 (91.7)	456 (87.0)	540 (88.7)	<0.001 ^1^
Clear cell carcinoma	7 (1.9)	13 (2.5)	10 (1.6)	
Mixed carcinoma	10 (2.8)	17 (3.2)	2 (0.3)	
Serous carcinoma	11 (3.0)	29 (5.5)	14 (2.3)	
Carcinosarcoma	0 (0.0)	4 (0.8)	10 (1.6)	
Other	2 (0.6)	5 (1.0)	33 (5.4)	
FIGO (2009)				
IA	124 (34.1)	220 (42.6)	301 (49.8)	<0.001
IB	100 (27.5)	93 (18.0)	84 (13.9)	
II	66 (18.1)	112 (21.7)	126 (20.9)	
IIIA	17 (4.7)	20 (3.9)	24 (4.0)	
IIIB	13 (3.6)	13 (2.5)	20 (3.3)	
IIIC or higher	44 (12.1)	58 (11.2)	49 (8.1)	
Grading				
G1	166 (46.5)	206 (40.2)	369 (62.6)	<0.001
G2	140 (39.2)	230 (44.9)	139 (23.6)	
G3	48 (13.4)	73 (14.3)	72 (12.2)	
Gx	3 (0.8)	3 (0.6)	9 (1.5)	
Type I	335 (92.0)	464 (87.9)	546 (89.5)	0.137
Type II	31 (8.5)	66 (12.5)	46 (7.5)	0.014

Data presented as n (%). ^1^ Comparisons were made with Pearson chi-square test or exact Fisher test.

**Table 3:** Overall survival differences between analyzed groups in 12-, 24-, and 36-month-long periods.

Time Period	12-Month Survival (%)	95% CI	24-Month Survival (%)	95% CI	36-Month Survival (%)	95% CI
2003–2008	89.6	[85.2–94.3]	83.3	[77.9–89.0]	78.0	[72.1–84.4]
2009–2014	88.1	[84.4–91.9]	76.9	[72.1–82.0]	66.2	[60.7–72.3]
2015–2020	92.8	[90.0–95.7]	83.5	[79.2–88.2]	69.9	[63.2–77.3]

Data presented as (%). CI—confidence interval.

**Table 4:** Overall survival differences between 2003–2008 and 2009–2014 groups.

Time Point (Months)	Group 2003–2008 Survival (%) [CI 95%]	Group 2009–2014 Survival (%) [CI 95%]
12	89.6 [85.2–94.3]	88.1 [84.4–91.9]
24	83.3 [77.9–89.0]	76.9 [72.1–82.0]
36	78.0 [72.1–84.4]	66.2 [60.7–72.3]
48	72.6 [66.2–79.6]	50.2 [43.7–57.7]
60	68.8 [62.2–76.2]	50.2 [43.7–57.7]

CI—confidence interval.

## Data Availability

Data are available upon request.
